# Supplementary material for: Glycolytic reliance promotes anabolism in photoreceptors
Source: eLife. 2017 Jun 9;6:e25946. doi: 10.7554/eLife.25946 (PMC5499945; doi:10.7554/eLife.25946)
Supplement: Supplementary file 1. — DOI: http://dx.doi.org/10.7554/eLife.25946.023 [file elife-25946-supp1.docx]

**Supplementary file 1.** qPCR analysis of target genes in isolated rod samples

| Sample | *Rpl13a* | *Actin* | *18S* | *Rho* | *PKM1* | *PKM2* | *LDHA* | *LDHB* |
| --- | --- | --- | --- | --- | --- | --- | --- | --- |
| #1 | 20.4 | 19.31 | >32 | 17.65 | 27.21 | 20.78 | 23.04 | >32 |
| #2 | 19.82 | 21.73 | >32 | 16.19 | 29.44 | 21.38 | 22.59 | >32 |

C_t_ values of gene targets for two isolated rod samples
