# Supplementary material for: Glycolytic reliance promotes anabolism in photoreceptors
Source: eLife. 2017 Jun 9;6:e25946. doi: 10.7554/eLife.25946 (PMC5499945; doi:10.7554/eLife.25946)
Supplement: Supplementary file 2. — DOI: http://dx.doi.org/10.7554/eLife.25946.024 [file elife-25946-supp2.docx]

**Supplementary file 2.**

shRNA-encoding constructs used and targeted regions in the cDNA

| sh name | Targeted sequence (5’🡪3’) |
| --- | --- |
| LDHAsh1 | GTTCCCAGTTAAGTCGTATAA |
| LDHAsh3 | CGTGAACATCTTCAAGTTCAT |
| LDHAsh4 | GTTCCCAGTTAAGTCGTATAA |
| PKM1+2sh | GCGGTGGCTCTGGATACAAAG |
| PKM2sh | CTACCACTTGCAGCTATTCGA |
