# Supplementary material for: Glycolytic reliance promotes anabolism in photoreceptors
Source: eLife. 2017 Jun 9;6:e25946. doi: 10.7554/eLife.25946 (PMC5499945; doi:10.7554/eLife.25946)
Supplement: Supplementary file 3. — DOI: http://dx.doi.org/10.7554/eLife.25946.025 [file elife-25946-supp3.docx]

| **Name** | **Vendor** | **Catalog Number** | **Application & Dilution** |
| --- | --- | --- | --- |
| LDHA | Sigma | AV54777 | IHC- 1:2250 |
| LDHA | Novus | NBP1-48336 | Western- 1:4000 |
| LDHB | Sigma | AV48210 | IHC- 1:800 |
| Glutamine Synthetase | Chemicon | MAB302 | IHC- 1:600 |
| SDH-A | CST | 11998 | IHC- 1:10 |
| PKM1 | CST | 7067 | IHC: 1:1200 |
| PKM2 | CST | 4053 | IHC: 1:1600, IP-1:50 |
| PKM1 | ProteinTech | 15821-1-AP | Western- 1:2000 |
| PKM2 | CST | 3198 | Western- 1:1000, IHC- 1:50 |
| PKM2 | ProteinTech | 15822-1-AP | IHC- 1:100 |
| GAPDH | Bethyl | A300-641A | Western- 1:4000 |
| P-Y105-PKM2 | CST | 3827 | Western- 1:750 |
| P-Y436- FRS2 | CST | 3861 | Western- 1:1000 |
| FRS2 | Santa Cruz | sc-17841 | Western- 1:500 |
| P-Y10- LDHA | CST | 8176 | Western- 1:750 |
| TIGAR | Pierce/ThermoFisher Scientific | PA1-41487 | Western- 1:1000 |
| PDHE1 | Abcam | ab110334 | IHC- 1:100 |
| FLAG | Sigma | F1804 | Western- 1:10,000 |
| PFKFB1 | Abcam | ab155564 | IHC- 1:1500 |
| PFKFB2 | Bethyl | A304-286A | IHC- 1:10000 |
| PFKFB4 | Abcam | ab71622 | IHC- 1:1250 |
| Isotype Control | CST | 3900 | IP-1:50 |
| COXIV | CST | 4850 | Western-1:1000 |
| Anti-rabbit conformation-specific IgG | CST | 3678 | Western-1:2000 |
| RHO |  | Clone 4d2 | IHC- 1:500 |

**Supplementary file 3** List of antibodies
