# Supplementary material for: Glycolytic reliance promotes anabolism in photoreceptors
Source: eLife. 2017 Jun 9;6:e25946. doi: 10.7554/eLife.25946 (PMC5499945; doi:10.7554/eLife.25946)
Supplement: Supplementary file 4. — DOI: http://dx.doi.org/10.7554/eLife.25946.026 [file elife-25946-supp4.docx]

**Supplementary file 4**

Probe sequences for *in situ* hybridization

| *Ptbp1* | ACAAAGGTCACAATGAGGCTCGGCAGCTGAGGATGGCCTGGACCCTCCGGGCTGGCCAGGGACATGGGAGAATCATGTCACTTGATTATAAGGGTGTAGGGGAGTGGCCCCAGCTCTTGCACAGCATTGGGGAGCCTATGTATAGGCCACCTGGCTCACCTGCAGGTCTAGAGACAACTGGTGGCCTTAGAGGGTGGGTGTACGGCCATGAGGCACGCTGGTTTGTAAGGCACAGAAAGCGGCTATGTTTGGGGCACCGCTGTGGCCTCACGCCTGAGCCCCTCAAGGCATGGTGCTGGGATTGACTGGGCCTCTGTGGTCCAAGCCATTTCCTGCCACTGCAGAGTGGGCTAGATTTAATTTTTTTAAAAACAGGTAAACTGATCTCTCTTTAAAAAAATAAAATCTCTGGTCTGTAAGGTCACTTCAGCTGCTTTTTAAAGTGGCTTTTCTGGAATGGTGGAAGCTGATGCAGGCCCACAGGCACCTAGATGGTGGACTTGGAAAAGGACACTCGCAGGTGGTGGTTCTCGCCCAGGTCATGGTTGTGCAGTTCAATCAGCGCCTGCAC |
| --- | --- |
| *Srsf3* | TCACTAACAGCCATAATCCTGAACTGGCTTCAACACCTAAAAAATGGCTAACTCTCATAAGTGACAAGCAAAGGGGAAAAAAAAATTCCTTCACTAATGTAGTGCTTCTCAGTTGTGTTTCTTACAAAGAATGAACTGGGTAACCAACTACTTTCCCAGGGATAAGAACAATGGCAACAGTGGTTCGCTCGACTGACTACTACACAATTATGCTAGTACTTTATTAATTCAAAAGCACTTTACAAGAAAATATAAGTATGGCTTCCAATAGGAATGTTTTACCTGGACTTGATACCAAGCTCACAGATTTTGAGTGTGCAAGGAAAAACAGGCTTTCAAGTTAAACAACAATTTGTAACCAAAACTTTAATCCCAAAGATTCTCACAAACATATTACAAATGACAGCATGAAAAACAATCTTGCACAGTAACTGAAAAGTTGAGCTCTACAATGTACCCTTAAACTGAAAGGACACTGGCATCTGAGTTTTACATTTCCAAATAAAGACGTTACAATAGCTCTGCATCCCCATAGAGAAACCAGGAAGGAACCCCTTTGCAAATCTTCAGTGTGGTTCCTACCCTTATCTGTTGGCTTGTGTTCACAGCAGCTCTTAAACACCGCTTTATCATTCCACCCACAGTGCCACAGTGTCTCAGCTACTAGAGGACTATAGCTTCTTTCAATTTCCGCCAGTTGCTTTATATCATTAGTACATCACACTTAAAACTGGGCAGGACCTAAGGTTTATTTCAGTCTATGAAAAACTAAAATTCAAAGAAAATTAAATCTTGCTTAAGGGAACATTGTAAAGTAACAATTCTTGATATCACGTGCCTCTTATGATCCATCTGAAAACCATAGAGAATTACATTGTTTTGTGTGTCACTGTTCCAAGAGACAAAACAAATTTAAACAGCTAAACCATCTTAAAAATGCACCAAGCTTATGAAGTCTCAAACAAAACTTTAATTTCCTGTACATACTCCTGTCAGATGAAGTTATTTCCTGTACACCACTTTTGCAAACTGGTCTTCTATTTCCTTTCATTTGACCTAGATCGGCTACGAGACCTAGAGAAGGATCGAGACGGCTTGTGA |
| *Pfkfb1* | GAGAACAAGGCCAGCAGTTCCTACAGGAGGAAGGTAGTGAGAACAGAGACAATCTGATCACTTGAAAAGGGCTCAGTAATGGGCAGGGACAGTGTCCAGGGCTTCCTCGGGTTCACGGGTGATGTCCACATTCTCAGGCTTGTCCCGGTGTGTGTTCACAGCCTCCACATTCAGGTAGATGGACTCCACTCTGCAGCCATAAGCCACAGGTGTGAGTTTGAGCACTGTATGCAGAGGACACTTGAGATAGGGCAGCTCATCTGAACTTTTATCCAGGAAGTATGCTAGAAGGCACCGCATGACAGCCTGGTGACAGATCACCAGTACATTTTCTTGCCGTTCTAGCTCCATTATAACTGGCTCAAGACGCTGAACCAGATCCTCATAGGACTCTCCCTTGGGATAGCGGTAACGATATTTATCCTGGTCCCGTAGTGCAAATTCTTCAGGGTAGTGTTCCTGAATTTCTTCATAAGTCATCTCTTCACAGACACCCGCATCAATCTCATTCAGGGCCTTCCACTGTTCATAGGGGACGCCTAGGGCTTCAGCTGTCTGAATGGTCCTCTTCATGTGGCTAGTCCACACCTTCAGGGAGCTGATGCTTTGAGACCGAATGAAGTTGGCCAGGGCATAGGCATACTGCTTGCCCCGAGCTGAGAGGCCAGAGTCACCTCCAATGCGGCCTCTAAGGTTGAGTTCACTCTCACCATGTCGGCATAGGTAGATAGATCGAGGTGTGACATGGATGTTCATGAGGTAGTAGGCTGTACGGCTTTGAACGTGGTCTTGCACTCGATTTACCATGTAGCGTGTGCCCACATCGAAGATCTTGATGTAGGACAGGTGGCTGTCCAATTCCTCATCCAAAGGCTGGTAGTTGATCTCATAGCACTCAATTCTCTTTAGAAAGTCTTCTAAAACCTTTTCTTGGTCACAGTCTATGTAATCAGGACTGCCGAGTTTCACTTGCTTGATGTTTTCTGCAATGATGTCTGGGTCATTACAAATAGACTCAATAAAGAAGACCTTGTAACCATGTTCTTTAGCAAACTGCAGAATCAATGATCGTCGTTCTCTGGTAGTGTTGGTGGCATCAAAAACCGCAACGTGACCTTCCTCACGGCTGAGATACTTATGGACATCCTTTAGGGCTGCTAGAGCACACTGCTTCCTGATAAGTTGGGCCTCCATGTTGTCTGGGCGAAAGAATTCATAGTTCCTGTAGCTCACTGCCTCTCGTCGATACTGACCTAAATTAAACACTTTAGTCGGTGTTCCTATCCAGTTGAGATAGCGTGTGAGCTTCGTAGAGATGTAGGTCTTGCCTCGAGCTGGTAAAC |
| *Pfkfb2* | CTCCCCACCAGGGTATCGGTACAGATACTTCTCTTGATCTCGGAGTGCAAATTCCTCTGGGTACCGTTGCTCAATCTCTGAATATGTCATCTCCTCACACACGCCAGCATCAATCTCATTGAGGATCTTCCACTGCTCATAGGTCACCCCAAGAGACTCAGCAGTCTGAATTGTCCTCTTCAACTGGCTCGTCCACACTTTAAGGTCCTGGATCTCCTGTTCCTCCAGAAACTTCTTCAGAGCATGAGCAAACTGCTTTCCTCGCACGGAGAGGCCAGAGTCACCCCCAATCTTCCCCAAAAGGTTGAACTCGCTCTCGCCGTGCCGGCAAAGGTAGATGGTGCGAGGATGGACGTGGATATTCATCAGGTAGTAGACAATCTTACTCTGGATGTAGTCCTGAACTCTGTTGACCAGGAATCTCTGGCCTACATTTATCACCTTTATGAAAGAAAGATCCTTATCATAGTTGTCTGGGTCAAGGGGCTGGTAAGTGACCTTGTAGCACTCAATTCTCTTCAGGAAGTCCTCCATCACATTCTCCCTATTCCTTTCGGGGTAGTCAGGGCTCGACACTTTTACCTC |
| *Pfkfb3* | CAATGCCATAAAGCCGACAAGGATGCTTTTCTACACTGGGGGTGACTCCTCAAGATGGCCAGCATGGTGGTATGAGGCATACAGCAGCAGACACTTGGCCAAAATATGCGGACAGCTCTGAGCAGGCGGCCTGACATGAGCCTTCCTTGGGCCACCGTCCCTTCTTGAACAATGGGAGTGAAGTTGAAGGCTCTTAGACTCCCCATCTTAAAGACACCCCAACTCCTCCCATTCAGAAGCTGCAAGCCTCCAAGGGCCAGCACCCTAGGTTCTTGAAGTGAGGTGTGGGGAGGGAGGGAAGGAAGGAGAGAAGAAGAGAAGGAAGGAGGGAGGAAGGAAGGAAGGAAGGAAGGAAGGAAGGAAGGAAGGAAGGAAAGAAAGAAGGAAAAGTGCAAACAAACAGCCAACAACAGAATCTACTCAGGTGCCTCCAGGCCCAAGGTGAAGAACAGAAGGCCTAAGGGACTCTGGGAAGAGGGGAGCCCTGGTTATTTTTTTCTGAAAAATCCTTCCTTCCACTGATACAACCAGTCTGTCCCTTAACTTGGGACCCAGAAGACATGTGGACCTGTAGAGGAGTCAGGGCAAGTCATTGCTCAGCTGACCAGTAGGCCTCAGCTCAAGGTTTCCTCAGGAGGAGAAATCCCATACCCAATGCAGCAATGCCCAAGCCTCTTGTCTCTGAGGCCTCCTCCTAGGCCAAGATGCATAAAAGCCACATGAGAATGTGCAACTTGTCCCTGAGCCTCTGGCAAGTGGGGTCTAACTCTGAATCTCAGGGTTCCCTGCCACTCTTATCTTCTGACTATCGACATGCCAGGCCAGGATCCAAAGAAACAAAACAAAACAGCGTGGCGGTGTCCAGGGCCCCCACTTAGGGGTCTGATCTCACCTCCTCTAGAACTATAGCCCTCCCCAAGCTTTGGAAGGGCCTGAGAGGTCAGACAGCCCTGGAAGTCGAAAGAGAACAGAGCGTAGGAAGGTCTGCTGCTCTACACAGGAAGCTGGGATCTGTACAACTTCCTAGTTGTCTTTGCCACCCCAACATTTCAGTGGGGGGAAGGGCTTCTACTCTTCAACATGCCGACCTCCATTCTCCCGAGTCCAGAAGTCCTTGCGATCACTTGGCCTCGAGAAGATGAGCAGGCACAAGGCAGGCTGTGGAAATGGAATGGAACCCACATCTCGGCTTTAGTGCTTCTGGGAAGAGTCGGCACCGCTCCGGGAGCTCTTCATGTT |
| *Pfkfb4* | GCAGGGCCACAGTGGAATGTCTTAGTGAGAGTTTTTTGTCTCAGTGTCTCATTTAAGGTTGCAAACGTCATGAAGCAGCCAGCCTGGTGAGTGGACTGCTCGAGTGCTGTAGCAGAGGGGCTGGCAGCAGAGCACATAGCCCAGGGATTCTCAGCAGCCATACCCAACCTGTCACTGGTGGTCAACAAAGAGGCCGGTGGGCCAGGCTGAAGGTTCTGAGTGTTGCTAGCCACCCATGGCTTCAGGAGTGTTCCTGCATGGTGGCACCATCAGTACAGCAAACACAAGATGGCCTGGAGTGACCCCTCTGACAGAGGTCAGGGTCACTGGTGAGCAGGGACTGTGACAAGGGCTTCCTCTGAAGGCCTGGATATGTCTACATTCTGAGGTCTGTCTCGGTGAGTGTTCACAGCTGCCACGTTCAGGAATATGGACTCCACTTTACAACCGTAAGCCACGGGTGTGAGCTTCAGGACTGTGTGCAAGGGGCATTTGAGGTAGGGCAGCTCTTCAGCTGCCTTGTCAAGGAAGTAGGCCAGGAGGCAGCGCATGACAGCCTGGTGGCAAATGACCAACACATTCTCCTGCCTCTCCAGTTCCATGATGACGGGCTCCAGCCGCTGCACCAGGTCTTCATAGGACTCACCCTTCGGGTACCGGTACCGGTACTTGTCCTGATCCCGCAGGGCAAACTCCAGCGGGTAGTGATCCTGGATTTCTTCGTAGGTCATTTCCTCACAGACGCCCGCATCGATCTCGTTGAGGACCTTCCACTGCTCATAAGGGACGCTCAGCGCCTCGGCTGTCTGGATCGTCCTCTTCATCTGGCTCGTCCAGACCTTCAGATCCTTAATGTTCTGGTCACTGATGAACTGGGCCAGATGCTTGGAAAACTCCCGGCCCCGGGGGGACAGTCCAGGATCCCCACCAATCCGGCCCTTGAGGTTTAGCTCGCTCTCCCCATGCCGGCAGAGGTAGATGGAGCGGGGTGTCACATGGATGTTCATGAGGTAATAAACGATGCGACTCTGGATGTGATCAGCAACACGGTTCACCACATAGCTCTGGCCCACGTCCATGATCTTGATGTAGGACAGATCC |
| *Ldha* | TATTTGGCATGACACTTGGGTGGTTGGTTCCATCATCCATATGCAGATCTTACATCTCACATAATATTGCAATGCACACTACAGACACATATAATATATAAAATATCCTGTACAACATTTATGCACAAGATATGCATCATGGACGTACACACTGGAGCCAGGTTATACGACTTAACTGGGAACTCGTCGGCCTAGGCATGTTTGGTGCAGGGCAGCAATGCAGCAGGCTCACAGGGGTAATCGAAGCCTGCAGTTGGCAGTGTGTCTCAGAGACAGTGGGACTGTCACACTAACCAGGTCACCACTACACAAGTACCACTTGGCAGCAGGGCAGAGCTGTGGGGAGTGAGATGTTTCCCCACACCATCTCAACACCACTGTACTAACCACAGCTCAGACGAGAAGGGTGTGGTCTGCCTAGAAGCCCTGCTGCAGCCTGGACAGTGAAGTGCTAGGACACGGGGAAGACTTTA |
| *Ldhb* Probe#1 | GCTGCAATTGCTAACTTTATTTGATCAGGAGCTCCTAGTGCAAACATCAAACAAGCCTGGGCTTTGATCTGTGAGCTCATATCACATGTCAGGGAAGAAGCAAACTGTGACCTACATACATGGCTGAAGACTAAAGGTTCATGGTCACATTGGAGGTTTGTGTTCTACAGCCTAGAGACTGGCAGTCACAGGTCTTTGAGGTCTTTCTGGATGTCCCACAGGGTGTCCGCACTCTTCCTGAGCTGAGCGACCTCATCGTCCTTCAGCTTCTGATTGATGACGCTGGTCA |
| *Ldhb*  Probe#2 | GCTGCAATTGCTAACTTTATTTGATCAGGAGCTCCTAGTGCAAACATCAAACAAGCCTGGGCTTTGATCTGTGAGCTCATATCACATGTCAGGGAAGAAGCAAACTGTGACCTACATACATGGCTGAAGACTAAAGGTTCATGGTCACATTGGAGGTTTGTGTTCTACAGCCTAGAGACTGGCAGTCACAGGTCTTTGAGGTCTTTCTGGATGTCCCACAGGGTGTCCGCACTCTTCCTGAGCTGAGCGACCTCATCGTCCTTCAGCTTCTGATTGATGACGCTGGTCAGCCCCCGAGCATTGAGGATGCACGGGAGACTGAGGAAGACTTCATTCTCAATGCCGTACATTCCCTTCACCATGGTAGACACGGGGTGAATCCGGGAGAGGTTTTTCAGCATGGACTCGATGA |
| *Pkm* M1-specific | CTGCCAGACTCCGTGAGAACTATCAAAGCTGCTGCTAAACACTTATAAGAGGCCTCCACGCTGCCCATGGCCATGGCCTCCATGAGGTCTGTGGAGTGACTGGAGGCTCGCACAAGCTCTTCAAACAGCAGACGGTGGAACATGGCTGCCTCAGCCTCCCGAGCTAT |
| *Pkm*  M2-specific | CTGCCAGACTTGGTGAGCACGATAATGGCCCCACTGCAGCACTTGAAGGAGGCCTCCACGGCACCCACGGCGGCAGCTTCTGTGGGGTCGCTGGTAATGGGCGCCAGGCGGCGGAGTTCCTCGAATAGCTGCAAGTGGTAGATGGCAGCCTCTGCCTCTCGGGCAAT |
