# Supplementary material for: Glycolytic reliance promotes anabolism in photoreceptors
Source: eLife. 2017 Jun 9;6:e25946. doi: 10.7554/eLife.25946 (PMC5499945; doi:10.7554/eLife.25946)
Supplement: Supplementary file 5. — DOI: http://dx.doi.org/10.7554/eLife.25946.027 [file elife-25946-supp5.docx]

**Supplementary file 5**

Primers for qPCR analysis

|  | Forward (5’-3’) | Reverse (5’-3’) |
| --- | --- | --- |
| *spry1* | ccttcaagtcttccaccagc | ggcctattaggacggtctcc |
| *spry2* | catcaggtcttggcagtgtg | agaggattcaagggagaggg |
| *spry4* | aggtcctgaactgcaccaag | ggggatttacacagacgtgg |
| *spred1* | agccatccaccacttgagtc | gaaagatgagcgaggagacg |
| *spred2* | cgctggagtcatctctggtc | ggacaggcgtctaggtgaac |
| *dusp6* | aggacaccacagtttttgcc | tatctcggatcactggagcc |
| *xlrs1* | ctatgccagctctccacttc | tcctcaccctcatcctctg |
| *mcar* | gatggacagggaggttcattac | ggtctggacaactgcaatct |
| *ldha* | gctccccagaacaagattacag | tcgcccttgagtttgtcttc |
| *ldhb* | acaagtgggtatggcatgtg | acaattttcggagtctggagg |
| *pkm2* | tcatgctgtctggagaaacag | gggtcgctggtaatggg |
| *18s* | ctgagaaacggctaccacatc | gcctcgaaagagtcctgtattg |
| *rpl13a* | cagtgcgccagaaaatgc | gtggagtcatactggaacatgtag |
| *ldhb* | ctgaccagcgtcatcaatca | agaaagacctcaaagacctgt |
| *actin* | cgccaccagttcgccatgga | tacagcccggggagcatcgt |
| *rho* | cagaaggcagagaaggaagtc | ctggtgggtgaagatgtagaag |
